# Supplementary material for: Explaining Global Turkey Biometric Diversity Through Principal Component Analysis
Source: Animals (Basel). 2025 Aug 28;15(17):2537. doi: 10.3390/ani15172537 (PMC12427549; doi:10.3390/ani15172537)
Supplement: Supplementary file 1 [file animals-15-02537-s001.zip › Supplementary Figure S1.pdf]

Supplementary Figure S1. Eigenvalues and percentage of cumulative explanatory power (up to 99%) offered by the principal components generated using all the variables in the study (morphometric measures and biometric indices).

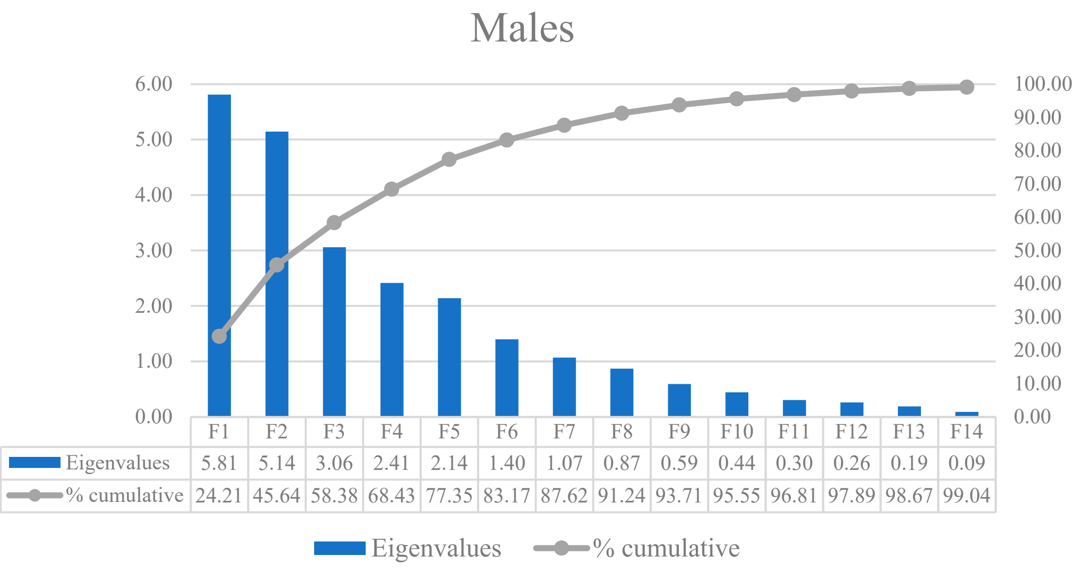

	F1	F2	F3	F4	F5	F6	F7	F8	F9	F10	F11	F12	F13	F14
Eigenvalues	5.81	5.14	3.06	2.41	2.14	1.40	1.07	0.87	0.59	0.44	0.30	0.26	0.19	0.09
% cumulative	24.21	45.64	58.38	68.43	77.35	83.17	87.62	91.24	93.71	95.55	96.81	97.89	98.67	99.04

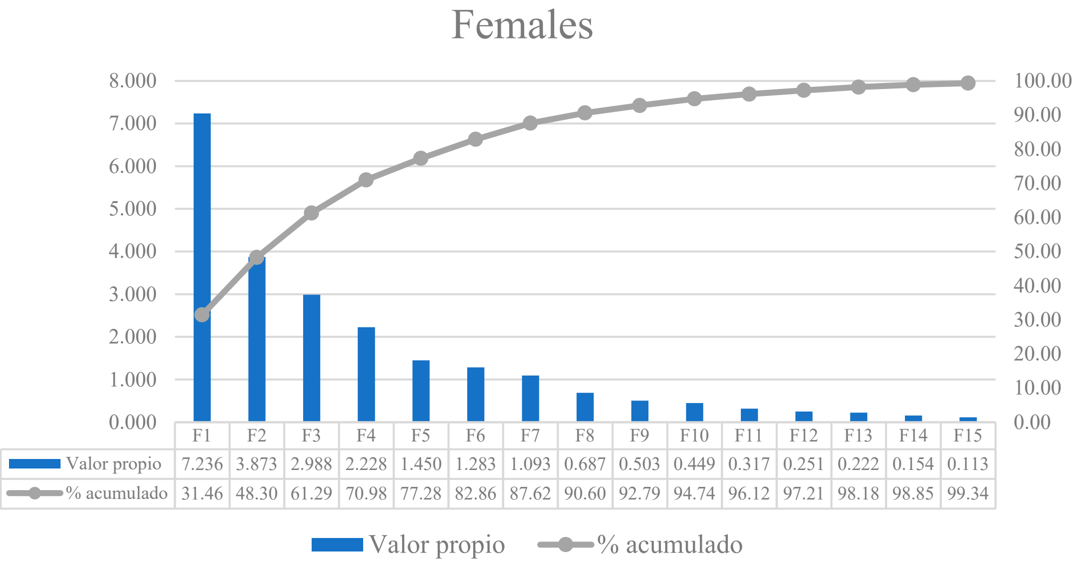

	F1	F2	F3	F4	F5	F6	F7	F8	F9	F10	F11	F12	F13	F14	F15
Valor propio	7.236	3.873	2.988	2.228	1.450	1.283	1.093	0.687	0.503	0.449	0.317	0.251	0.222	0.154	0.113
% acumulado	31.46	48.30	61.29	70.98	77.28	82.86	87.62	90.60	92.79	94.74	96.12	97.21	98.18	98.85	99.34
