# Supplementary material for: Explaining Global Turkey Biometric Diversity Through Principal Component Analysis
Source: Animals (Basel). 2025 Aug 28;15(17):2537. doi: 10.3390/ani15172537 (PMC12427549; doi:10.3390/ani15172537)
Supplement: Supplementary file 1 [file animals-15-02537-s001.zip › Supplementary Table S1.pdf]

Supplementary Table S1. Sexual dimorphism in the different morphometric parameters for each genotype measured in proportions (mean values for male/female) and in units (mean values for male – female), in brackets.

	W (*Kg*)	WS (*cm*)	BoL (*cm*)	SkL (*cm*)	SkW (*cm*)	BeL (*cm*)	BeW (*cm*)	SnL (*mm*)	NL (*cm*)	BrC (*cm*)	KL (*cm*)	ThL (*cm*)	TaL (*cm*)	TaD (*mm*)	TaW (*mm*)	MFL (*cm*)	Average
**NDI**	2.24 *(3.74)*	1.21 *(10.67)*	1.18 *(9.72)*							1.31 *(13.07)*	1.41 *(5.16)*		1.25 *(2.45)*		1.54 *(3.62)*		**1.45**
**KEN**	2.12 *(3.81)*												1.25 *(3.44)*				**1.69**
**BRI**	2.05 *(2.80)*	1.17 *(8.60)*	1.22 *(11.51)*							1.31 *(12.60)*	1.29 *(3.50)*		1.21 *(1.97)*		1.45 *(3.40)*		**1.39**
**TUN**	1.75 *(2.69)*		1.21 *(5.92)*			1.14 *(0.36)*					1.27 *(3.15)*	1.24 *(4.36)*	1.26 *(2.81)*				**1.31**
**ZAG**	1.70 *(2.69)*		1.24 *(5.72)*	1.20 *(1.98)*	1.21 *(0.72)*	1.10 *(0.33)*					1.31 *(3.81)*	1.21 *(3.89)*	1.25 *(3.10)*				**1.28**
**DAL**	1.69 *(2.92)*			1.18 *(1.73)*	1.18 *(0.67)*						1.39 *(4.70)*	1.19 *(3.32)*	1.30 *(3.64)*				**1.32**
**MEX**	1.65 *(2.11)*	1.20 *(11.40)*	1.09 *(4.23)*			1.07 *(0.33)*		2.59 *(5.10)*	1.16 *(3.89)*	1.16 *(7.54)*		1.16 *(2.06)*	1.21 *(2.43)*		1.71 *(6.35)*		**1.42**
**GHA**	1.64 *(2.35)*		1.21 *(6.01)*	1.51 *(2.44)*			1.14 *(0.36)*				1.37 *(3.88)*	1.22 *(3.93)*	1.27 *(2.85)*				**1.34**
**NOR**	1.63 *(2.17)*		1.13 *(4.02)*							1.25 *(10.93)*		1.20 *(3.35)*	1.22 *(2.64)*				**1.29**
**CAM**	1.62 *(3.04)*		1.17 *(9.18)*							1.27 *(13.32)*			1.25 *(2.91)*		1.01 *(0.20)*		**1.26**
**MAM**	1.62 *(2.08)*		1.12 *(3.61)*							1.23 *(9.90)*		1.16 *(2.79)*	1.23 *(2.69)*				**1.27**
**GUA**	1.46 *(1.17)*		1.18 *(8.28)*	1.13 *(1.26)*	1.14 *(0.52)*			3.78 *(5.43)*									**1.74**
**BAN**	1.41 *(1.65)*		1.09 *(2.72)*			1.26 *(1.12)*						1.25 *(3.29)*	1.13 *(1.29)*				**1.23**
**COM**	1.36 *(2.89)*	1.09 *(6.62)*	1.11 *(6.33)*	0.83 *(-2.00)*	0.84 *(-0.68)*	1.14 *(0.78)*	1.24 *(0.56)*		1.09 *(2.08)*	1.05 *(2.96)*	1.10 *(1.81)*	1.11 *(2.41)*	1.22 *(3.19)*	1.12 *(2.50)*	1.10 *(1.43)*	1.22 *(1.78)*	**1.11**
**NIG**	1.28 *(0.95)*	1.15 *(9.33)*	1.14 *(6.37)*	1.40 *(2.68)*		1.08 *(0.41)*			1.25 *(4.80)*	1.14 *(5.84)*	1.27 *(3.49)*	1.19 *(3.07)*	1.23 *(2.11)*			1.11 *(0.82)*	**1.21**
**Average**	**1.68**	**1.16**	**1.16**	**1.21**	**1.09**	**1.13**	**1.19**	**3.19**	**1.17**	**1.22**	**1.30**	**1.19**	**1.23**	**1.12**	**1.36**	**1.17**	

Where “W” represents weight, “WS” wing span, “BoL” body length, “SkL” skull length, “SkW” skull width, “BeL” beak length, “BeW” beak width, “SnL” snood length, “NL” neck length, “BrC” breast circumference, “KL” keel length, “ThL” thigh length, “TaL” tarsus length, “TaD” tarsus depth, “TaW” tarsus width and “MFL” middle finger length.
